# Providing culturally responsive care in a pediatric setting: are our trainees ready?

**DOI:** 10.1186/s12909-023-04651-0

**Published:** 2023-09-20

**Authors:** Anna Chen, Zachary Blatman, Amy Chan, Anna Hossain, Chavon Niles, Adelle Atkinson, Indra Narang

**Affiliations:** 1 University of Toronto Temerty Faculty of Medicine, 1 King’s College Cir, Toronto, ON M5S 1A8 Canada; 2 Division of Respiratory Medicine, The Hospital for Sick Children, 555 University Ave, Toronto, ON M5G 1X8 Canada; 3 Research Institute Equity, Diversity & Inclusion, The Hospital for Sick Children, 555 University Ave, Toronto, ON M5G 1X8 Canada; 4 Department of Pediatrics, The Hospital for Sick Children, 555 University Ave, Toronto, ON M5G 1X8 Canada; 5 Division of Immunology and Allergy, The Hospital for Sick Children, 555 University Ave, Toronto, ON M5G 1X8 Canada

**Keywords:** Medical education, Pediatrics, Survey, Equity, diversity, and inclusion, Culturally responsive care

## Abstract

**Background:**

Extensive data consistently demonstrates inequities in access and delivery of healthcare for patients from historically marginalized populations, resulting in poorer health outcomes. To address this systemic oppression in healthcare, it is necessary to embed principles of equity, diversity, and inclusion (EDI) at an early stage within medical education. This study aimed to assess pediatric trainees’ perceived interest in EDI curricula as well as their confidence in applying this knowledge to provide culturally responsive care.

**Methods:**

An anonymous online survey was distributed to pediatric trainees at the University of Toronto. Closed-ended questions used a Likert scale to assess respondents’ confidence and interest in providing culturally responsive care to patients. Open-ended questions explored trainees’ perceptions of effective EDI learning modalities. A mixed methods approach was utilized, where quantitative data was summarized using descriptive statistics and descriptive content analysis was used to highlight themes within qualitative data.

**Results:**

116 pediatric trainees completed the survey, of which 72/116 (62%) were subspecialty residents/fellows and 44/116 (38%) were core residents. 97% of all responses agreed or strongly agreed that it was important to learn about providing culturally responsive care to patients from historically marginalized communities; however, many trainees lacked confidence in their knowledge of providing culturally responsive care (42%) and applying their knowledge in clinical practice (47%). Respondents identified direct clinical exposure through rotations, immersive experiences, and continuity clinics as effective EDI teaching modalities. Identified barriers included time constraints in the clinical environment, burnout, and lack of exposure to diverse patient populations.

**Conclusion:**

Most pediatric trainees want to provide culturally responsive care to patients from historically marginalized communities, but do not feel confident in their knowledge to do so. Trainees value learning about EDI through direct clinical exposure and immersive experiences, rather than didactic lectures or modules. These study findings will be utilized to develop and implement an enhanced EDI education curriculum for pediatric trainees at the University of Toronto and other postgraduate residency programs.

## Background

Racism, sexism, and ableism have been pervasive within medical education for centuries. From the misconstruction of race as a biological or genetic risk factor, to the paucity of education around historical injustices and healthcare disparities, there continues to be a growing need to integrate a health equity lens within medical education. The effects of systemic oppression within healthcare are real, widespread, and often trivialized [1, 2]. Extensive data demonstrates poorer health outcomes and negative treatment for patients who identify as Indigenous, Black, racialized, 2SLGBTQIA+, disabled, economically disadvantaged, or belonging to a minoritized faith group [3–7]. Recognizing this, medical educators must ensure that trainees gain awareness about systemic oppression within the healthcare system and acquire the skills needed to deliver culturally responsive care.

“Cultural competence” is defined as “an ability to learn from and respectfully relate to other cultural backgrounds, heritages, and traditions” [8]. Expanding on this concept, “culturally responsive care” is a person-centered framework that encourages healthcare providers to respond to every patient’s unique social and cultural identities, and engage in continuous self-reflection throughout every clinical interaction [9]. Teaching culturally responsive principles to healthcare providers promotes excellence in clinical care [10, 11]. However, there continues to be a lack of awareness regarding systemic inequities that exist within healthcare. Shepherd et al. explored how American healthcare professionals (n = 56) perceived cross-cultural educational models, such as cultural competence [12]. Though many healthcare providers believed they had the knowledge to treat patients from historically marginalized groups, there was a disconnect between perceived and actual knowledge of the structural imbalances within the healthcare system. While most participants recognized the importance of a patient’s culture, only half of respondents acknowledged that cultural competence would improve care [12]. This highlights the need for further education on culturally responsive care.

Many postgraduate medical programs have introduced equity, diversity, and inclusion (EDI) content into the formal academic curriculum [13]. In the University of Toronto’s (UofT) postgraduate Core Pediatric Residency Program, EDI training has been offered by the Department of Pediatrics at The Hospital for Sick Children. In the 2020-21 year, core pediatric residents participated in a foundational Allyship workshop, anti-Black and anti-Indigenous racism training, a seminar on how to address microaggressions, and a session on Indigenous health [14, 15]. However, no research has specifically examined pediatric trainees’ interest in EDI curricula and confidence in applying this knowledge to providing culturally responsive care to marginalized patient populations. Study findings will help inform educational strategies required to develop a more comprehensive and integrative EDI curriculum that prepares trainees to deliver culturally responsive clinical care.

## Methods

### Study design

In this cross-sectional needs assessment study, an anonymous survey was sent to assess the interest and confidence levels of pediatric core residents and subspecialty residents/fellows at UofT in providing culturally responsive care to patients. The survey was designed at The Hospital for Sick Children by the Associate Chair of EDI in the Department of Pediatrics and the Program Manager of EDI as well as medical student and graduate student research team members. This current project was designed in response to written and verbal feedback received from trainees during previous Allyship workshops at the Hospital for Sick Children. The results, which were recently published, highlighted a lack of training and knowledge to embed EDI practices into everyday clinical care [14]. Survey questions were also developed with adherence to the Ontario Anti-Racism Data Standards [16]. Survey creators had knowledge and training in EDI and medical education, and included those from historically underrepresented groups (e.g., race, gender, sexual orientation) and those with privilege (e.g., race, gender, sexual orientation, socioeconomic status, ability).

Recognizing that language evolves, the study defined historically marginalized communities as individuals who identify as Indigenous, Black, racialized, 2SLGBTQIA+, disabled, economically disadvantaged, or belonging to a minoritized faith group. Marginalization refers to a social process in which individuals experience reduced access to power and resources due to aspects of their identity such as race, religion, gender, sexual orientation, class, or disability status [17]. The term “historically marginalized” was intentionally utilized to recognize the history of systemic practices that privileged some groups and disadvantaged others in North America [17]. A health equity framework, which acknowledges the complex interplay between individuals and their environments and their influence on health outcomes, informed survey design and analysis [18].

The survey contained participant demographic information, as well as six closed-ended statements and six open-ended questions (Table 1). Five closed-ended statements used a five-point Likert Scale from Strongly Disagree [1] to Strongly Agree [5] to answer questions related to each of the seven populations of interest (i.e., Indigenous, Black, racialized, 2SLGBTQIA+, disabled, economically disadvantaged, and belonging to a minoritized faith group). One closed-ended statement used an eight-point Likert Scale from most effective [1] to least effective [8] to rank teaching modalities. Open-ended questions asked respondents to describe previous EDI education, perceptions of effective teaching modalities, and enabling factors to learning about culturally responsive care.

A survey draft was shared with the UofT Department of Pediatrics’ Core Residency EDI committee for additional feedback prior to distribution. Research Ethics Board approval was obtained from the Hospital for Sick Children, Toronto, Canada (Research Ethic Board #1,000,077,314). Table 1 includes the survey questions sent to eligible participants.

**Table 1:** Survey questions

Closed-Ended Statements:	Open-Ended Questions:
• In the postgraduate pediatrics curriculum, it is important to learn about providing culturally responsive care to children and youth who identify as (1) Indigenous, (2) Black, (3) racialized, (4) 2SLGBTQIA+, (5) having a disability, (6) economically disadvantaged, or (7) belonging to a minoritized faith group. • In the postgraduate pediatrics curriculum, I am interested in learning about providing culturally responsive care to children and youth who identify as (1) Indigenous, (2) Black, (3) racialized, (4) 2SLGBTQIA+, (5) having a disability, (6) economically disadvantaged, or (7) belonging to a minoritized faith group. • I am confident about my knowledge in providing culturally responsive care to children and youth who identify as (1) Indigenous, (2) Black, (3) racialized, (4) 2SLGBTQIA+, (5) having a disability, (6) economically disadvantaged, or (7) belonging to a minoritized faith group. • In a clinical setting, I feel confident applying my knowledge to provide culturally responsive care to children and youth who identify as (1) Indigenous, (2) Black, (3) racialized, (4) 2SLGBTQIA+, (5) having a disability, (6) economically disadvantaged, or (7) belonging to a minoritized faith group. • In a clinical setting, I feel confident with addressing my own biases while providing culturally responsive care to children and youth who identify as (1) Indigenous, (2) Black, (3) racialized, (4) 2SLGBTQIA+, (5) having a disability, (6) economically disadvantaged, or (7) belonging to a minoritized faith group. • Please rank the following learning modalities from most effective (1) to least effective (8) in teaching residents and fellows culturally responsive ways of providing care to pediatric patients: (1) Continuity clinic with a provider focusing on a specific underserved population, (2) Didactic lectures, (3) Direct clinical exposure through a clinical rotation, (4) Immersive experiences, (5) Internet modules, (6) Patient panels, (7) Small group workshops led by leaders of underserviced populations, (8) Standardized patients in clinical skills.	• Please describe why you think the top ranked teaching modality (1) in the previous question is the most effective. • Please list other effective teaching modalities. • What do you think are factors that encourage residents and fellows to learn about providing culturally responsive care to pediatric patients? • What do you think are factors that prevent residents and fellows from learning about providing culturally responsive care to pediatric patients? • Please describe the education you received on providing culturally responsive care during medical school. • Please describe any additional education or training you received on providing culturally responsive care outside of medical school.

### Study recruitment process

Trainees were eligible to participate in the study if they were enrolled in any year of UofT’s Core Pediatric Residency Training Program or any UofT Pediatric Subspecialty Residency or Fellowship Program during the July 1, 2021-June 30, 2022 academic year. There were no specific exclusion criteria. Eligible participants were recruited to complete an anonymous survey online. Informed consent was provided in accordance with institutional policies. Trainees were given a gift card for voluntary completion of the survey.

The survey link was sent via emails to pediatric trainees’ The Hospital for Sick Children internal email addresses through coordination with the former Core Pediatric Residency Program Director (A.A.). Follow-up emails were sent by the Principal Investigators (I.N., A.A.). Participants were recruited from September 2, 2021 until January 18, 2022.

### Data Analysis

A mixed methods approach was utilized to analyze quantitative and qualitative data. Demographic data was summarized using descriptive statistics. Results of Likert-scale questionnaire data were summarized using frequencies. Answers of “strongly agree” and “agree” were considered positive responses, while answers of “disagree” and “strongly disagree” were considered negative responses. Teaching modalities were ranked 1 to 8 by respondents, and analyzed using a weighted-rank score. The most effective teaching modality (ranked first) would receive a score of 8, while the least effective teaching modality (ranked eighth) would receive a score of 1. We then summated the total scores and visualized the data utilizing a bar graph.

Transcripts and open-ended survey responses were analyzed iteratively using descriptive content analysis. Data was first descriptively analyzed by two members of the research team (Z.B. and A.C.) who assigned codes to important and relevant findings. Codes were subsequently grouped into categories and overarching themes. Findings were shared with the broader research team (A.C., A.H., A.A., I.N.), who met throughout the analysis process to reach consensus on data interpretation.

## Results

### Participant demographics

A total of 116 pediatric trainees completed the survey, including 44 pediatric core residents and 72 subspecialty residents/fellows. All 81 core residents and 325 subspecialty residents/fellows received the anonymous survey link. Thus, the response rates were 54% (44/81) for core residents, and 22% (72/325) for subspecialty residents/fellows. Among core residents, respondents were distributed across all four years of training (34% Postgraduate Year (PGY)-1, 25% PGY-2, 21% PGY-3, and 21% PGY-4). Of respondents, 83/116 (72%) identified as female and 33/116 (28%) identified as male. The majority of respondents identified as White [60/116 (52%)] and heterosexual [105/116 (91%)]. Please refer to Table 2 for full demographic data.

**Table 2:** Participant demographics

	Core Residents n (%)	Subspecialty Residents/Fellows n (%)	Total n (%)
**Year of Training**			
PGY-1	15 (34.1)		15 (12.9)
PGY-2	11 (25)		11 (9.5)
PGY-3	9 (20.5)		9 (7.8)
PGY-4	9 (20.5)		9 (7.8)
Subspecialty Resident/Fellow		72 (100)	72 (62)
**Location of Undergraduate Medical Education**			
Canada	35 (79.5)	25 (34.7)	60 (51.7)
International	9 (20.5)	47 (65.3)	56 (48.3)
**Identification as Indigenous (Original People) of Turtle Island (North America which includes United States and Canada)**			
Yes (First Nations, Métis, Inuit, not listed)	0 (0)	0 (0)	0 (0)
No	44 (100)	72 (100)	116 (100)
**Race**			
Indigenous	0 (0)	0 (0)	0 (0)
Black	1 (2.3)	3 (4.2)	4 (3.4)
East Asian	5 (11.3)	7 (9.7)	12 (10.3)
Latino/Latina/Latinx	0 (0)	6 (8.3)	6 (5.2)
South Asian	5 (11.4)	10 (13.9)	15 (12.9)
Southeast Asian	1 (2.3)	0 (0)	1 (0.9)
West Asian or Middle Eastern	5 (11.3)	7 (9.7)	12 (10.3)
White	24 (54.5)	36 (50)	60 (51.7)
Mixed Race*	2 (4.5)	2 (2.7)	4 (3.4)
Prefer not to answer	1 (2.3)	1 (1.4)	2 (1.7)
**Religion**			
Agnostic	6 (13.6)	10 (13.9)	16 (13.8)
Atheist	9 (20.5)	7 (9.7)	16 (13.8)
Buddhist	0 (0)	2 (2.8)	2 (1.7)
Christian	11 (25)	22 (30.6)	33 (28.4)
Hindu	1 (2.3)	5 (6.9)	6 (5.2)
Islam	5 (11.4)	7 (9.7)	12 (10.3)
Jewish	8 (18.2)	15 (20.8)	23 (19.8)
Sikh	2 (4.5)	0 (0)	2 (1.7)
Pagan	0 (0)	0 (0)	0 (0)
Wicca	0 (0)	0 (0)	0 (0)
Prefer to Describe: Baha’i	1 (2.3)	0 (0)	1 (0.9)
Prefer to Describe: No strong beliefs	1 (2.3)	0 (0)	1 (0.9)
Multiple Religious Beliefs**	0 (0)	2 (2.8)	2 (1.7)
Prefer not to answer	2 (4.5)	2 (2.8)	4 (3.4)
**Gender Identity**			
Female	34 (77.3)	49 (68.1)	83 (71.6)
Male	10 (22.7)	23 (31.9)	33 (28.4)
Genderqueer/Gender nonconforming	0 (0)	0 (0)	0 (0)
Two-Spirit	0 (0)	0 (0)	0 (0)
Prefer not to answer	0 (0)	0 (0)	0 (0)
**Sexual Orientation**			
Gay	1 (2.3)	1 (1.4)	2 (1.7)
Lesbian	0 (0)	0 (0)	0 (0)
Bisexual	3 (6.8)	4 (5.6)	7 (6.0)
Heterosexual/Straight	39 (88.6)	66 (91.7)	105 (90.5)
Two-Spirit	0 (0)	0 (0)	0 (0)
Queer	1 (2.3)	0 (0)	1 (0.9)
Prefer not to answer	0 (0)	1 (1.4)	1 (0.9)
**Disability**			
Yes	0 (0)	2 (2.8)	2 (1.7)
No	44 (100)	70 (97.2)	114 (98.3)
Prefer not to answer	0 (0)	0 (0)	0 (0)

PGY = Postgraduate Year

*Mixed Race: 1 respondent identified as East Asian/White, 1 respondent identified as Latino/White, and 1 respondent identified as West Asian or Middle Eastern/White. 1 respondent identified as mixed race, but did not further specify

**Multiple Religious Beliefs: 1 respondent identified as Christian/Atheist and 1 respondent identified as Muslim/Agnostic

### Participant interest and confidence in providing culturally responsive care

In considering aggregate responses, i.e., the calculated average of all responses, nearly all respondents indicated importance (97%) and interest (95%) in learning about providing culturally responsive care to children and youth from the survey’s identified historically marginalized communities. More specifically, respondents expressed importance and interest as follows: Indigenous [112/116 (97%) importance, 111/116 (96%) interest], Black [112/116 (97%) importance, 110/116 (95%) interest], racialized [112/115 (97%) importance, 112/116 (97%) interest], 2SLGBTQIA+ [110/115 (96%) importance, 107/116 (92%) interest], disabled [115/116 (99%) importance, 112/116 (97%) interest), economically disadvantaged [113/116 (97%) importance, 109/114 (96%) interest], and belonging to a minoritized faith group (107/115 (93%) importance, 105/114 (92%) interest]. In contrast, many pediatric trainees lacked confidence in their knowledge of providing culturally responsive care to children and youth from historically marginalized communities (42% agreed or strongly agreed when aggregating responses from all the survey’s identified historically marginalized communities) and applying their knowledge in clinical practice (47% agreed or strongly agreed when aggregating responses from all the survey’s identified historically marginalized communities): Indigenous [33/116 (29%) confidence in knowledge, 39/115 (34%) confidence in knowledge application], Black [42/116 (36%) confidence in knowledge, 50/116 (46%) confidence in application], racialized (44/116 (38%) confidence in knowledge, 49/116 (42%) confidence in application], 2SLGBTQIA+ [61/116 (53%) confidence in knowledge, 61/114 (54%) confidence in application], disabled [65/115 (57%) confidence in knowledge, 69/116 (59%) confidence in application], economically disadvantaged [58/116 (50%) confidence in knowledge, 65/116 (56%) confidence in application], and belonging to a minoritized faith group [38/116 (33%) confidence in knowledge, 46/116 (40%) confidence in application]. Table 3 summarizes this data.

**Table 3:** Pediatric trainees’ confidence and interest in providing culturally responsive care

Question	Strongly Agree n (%)	Agree	Neutral	Disagree	Strongly Disagree	Respondents (n)
**In the postgraduate pediatrics curriculum, it is important to learn about providing culturally responsive care to**:						
Indigenous children/youth (First Nations, Inuit, and Métis)	90 (77.6)	22 (19)	3 (2.6)	1 (0.9)	0 (0)	116
Black children/youth	88 (75.9)	24 (20.7)	3 (2.6)	1 (0.9)	0 (0)	116
Racialized children/youth	86 (74.8)	26 (22.6)	2 (1.7)	1 (0.9)	0 (0)	115
2SLGBTQIA + children/youth	86 (74.8)	24 (20.9)	3 (2.6)	2 (1.7)	0 (0)	115
Children/youth with a disability/disabilities	97 (83.6)	18 (15.5)	0 (0)	1 (0.9)	0 (0)	116
Economically disadvantaged children/youth	87 (75)	26 (22.4)	2 (1.7)	1 (0.9)	0 (0)	116
Children/youth from a minoritized faith group	80 (69.6)	27 (23.5)	5 (4.3)	3 (2.6)	0 (0)	115
**In the postgraduate pediatrics curriculum, I am interested in learning about providing culturally responsive care to**:						
Indigenous children/youth (First Nations, Inuit, and Métis)	79 (68.1)	32 (27.6)	4 (3.4)	1 (0.9)	0 (0)	116
Black children/youth	79 (68.1)	31 (26.7)	6 (5.2)	0 (0)	0 (0)	116
Racialized children/youth	76 (65.5)	36 (31.0)	4 (3.4)	0 (0)	0 (0)	116
2SLGBTQIA + children/youth	78 (67.2)	29 (25)	8 (6.9)	1 (0.9)	0 (0)	116
Children/youth with a disability/disabilities	79 (68.1)	33 (28.4)	4 (3.4)	0 (0)	0 (0)	116
Economically disadvantaged children/youth	77 (67.5)	32 (28.1)	5 (4.4)	0 (0)	0 (0)	114
Children/youth from a minoritized faith group	69 (60.5)	36 (31.6)	7 (6.1)	2 (1.8)	0 (0)	114
**I am confident about my knowledge in providing culturally responsive care to**:						
Indigenous children/youth (First Nations, Inuit, and Métis)	3 (2.6)	30 (25.9)	37 (31.9)	39 (33.6)	7 (6.0)	116
Black children/youth	7 (6.0)	35 (30.2)	42 (36.2)	30 (25.9)	2 (1.7)	116
Racialized children/youth	6 (5.2)	38 (32.8)	36 (31.0)	33 (28.4)	3 (2.6)	116
2SLGBTQIA + children/youth	11 (9.5)	50 (43.1)	24 (20.7)	24 (20.7)	7 (6.0)	116
Children/youth with a disability/disabilities	14 (12.2)	51 (44.3)	29 (25.2)	20 (17.4)	1 (0.9)	115
Economically disadvantaged children/youth	13 (11.2)	45 (38.8)	32 (27.6)	22 (19.0)	4 (3.4)	116
Children/youth from a minoritized faith group	7 (6.0)	31 (26.7)	42 (36.2)	33 (28.4)	3 (2.6)	116
**In a clinical setting, I feel confident applying my knowledge to providing culturally responsive care to**:						
Indigenous children/youth (First Nations, Inuit, and Métis)	4 (3.5)	35 (30.4)	45 (39.1)	27 (23.5)	4 (3.5)	115
Black children/youth	8 (6.9)	42 (36.2)	42 (36.2)	23 (19.8)	1 (0.9)	116
Racialized children/youth	8 (6.9)	41 (35.3)	38 (32.8)	27 (23.3)	2 (1.7)	116
2SLGBTQIA + children/youth	10 (8.8)	51 (44.7)	31 (27.2)	16 (14.0)	6 (5.3)	114
Children/youth with a disability/disabilities	8 (6.9)	61 (52.6)	35 (30.2)	11 (9.5)	1 (0.9)	116
Economically disadvantaged children/youth	11 (9.5)	54 (46.6)	32 (27.6)	18 (15.5)	1 (0.9)	116
Children/youth from a minoritized faith group	8 (6.9)	38 (32.8)	44 (37.9)	25 (21.6)	1 (0.9)	116
**In a clinical setting, I feel confident with addressing my own biases while providing culturally responsive care to**:						
Indigenous children/youth (First Nations, Inuit, and Métis)	14 (12.1)	58 (50)	34 (29.3)	9 (7.8)	1 (0.9)	116
Black children/youth	18 (15.7)	51 (44.3)	34 (29.6)	11 (9.6)	1 (0.9)	115
Racialized children/youth	18 (15.8)	49 (43.0)	34 (29.8)	11 (9.6)	2 (1.8)	114
2SLGBTQIA + children/youth	13 (11.2)	56 (48.3)	35 (30.2)	10 (8.6)	2 (1.7)	116
Children/youth with a disability/disabilities	18 (15.5)	55 (47.4)	33 (28.4)	9 (7.8)	1 (0.9)	116
Economically disadvantaged children/youth	17 (14.7)	56 (48.3)	32 (27.6)	11 (9.5)	0 (0)	116
Children/youth from a minoritized faith group	15 (12.9)	51 (44.0)	40 (34.5)	9 (7.8)	1 (0.9)	116

### Effective learning modalities

**Fig. 1:**
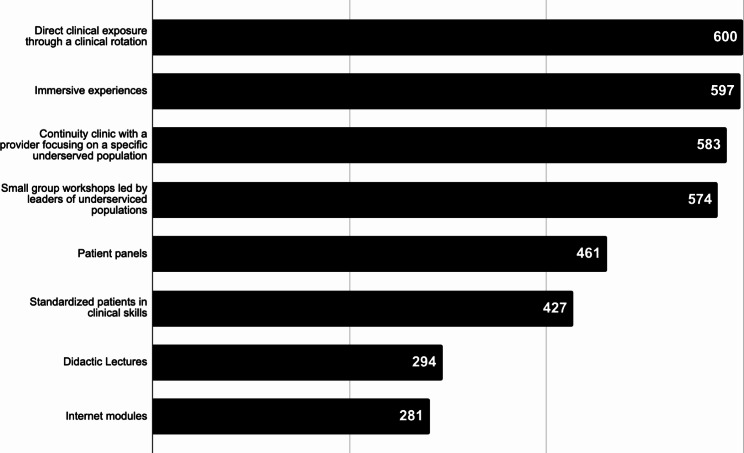
Pediatric trainees’ perceptions of effective teaching modalities for learning how to provide culturally responsive care. Values represent a weighted-rank score of student preferences, with higher values representing teaching modalities with higher perceived effectiveness.

Learning modalities perceived as most effective included direct clinical exposure through a clinical rotation, immersive experiences, and continuity clinics with a provider focusing on a specific underserved population. Learning modalities perceived as least effective included internet modules, didactic lectures, and standardized patients in clinical skills. Figure 1 summarizes the data on respondents’ perceptions of effective strategies to learn about EDI.

### Qualitative themes

Four key themes emerged from the qualitative data collection: (1) hands-on opportunities as effective teaching modalities; (2) enabling factors to learning about culturally responsive care; (3) barriers to learning about culturally responsive care; and (4) medical school education on culturally responsive care. All themes with example quotations are included in Table 4.

**Table 4:** Pediatric trainees’ perceptions of facilitators and barriers to learning about providing culturally responsive care

Themes	Quotations
Hands-on opportunities as effective teaching modalities	*“Continuity allows you to learn over time, apply what you have learned, and address your own biases along the way. A one time encounter would not allow this.”* *“The ability to follow through each experience, understand the system, learn how you can make a change based on the position you are in. Discussions and teaching with staff afterwards.”* *“By becoming immersed in various communities, I believe it will allow learners to get a deeper understanding of the specific population and have a chance to ask questions and figure out how to be more culturally sensitive.”* *“The underserved population has the best perspective for providing direct feedback. I have had nonjudgmental feedback provided by such leaders in the past and it has had profound, lasting impacts on how to address internalized biases.”* *“Opportunity to learn, ask questions, get feedback, see examples all in real time and with real patients. These are effective experiences for helping shift our clinical practice.”* *“I come from Australia, where we had “indigenous health workers”. Their role was to facilitate health care with indigenous families, they provided invaluable insight into how to provide culturally appropriate care and address barriers to health care. Working along side them was one of the most valuable learning experiences I had.”* *“In my experience, the more “real life” the learning opportunities are, the more helpful. So much of what I’ve learned about providing culturally sensitive care is through my diabetes clinic. Most families are of lower SES and that will affect their ability to afford certain diabetes related technology, healthy foods, etc. some cultures also emphasize the independence of youth earlier than others which may result in a child having more responsibility at the same developmental age when compared to a child from a different culture or religion. Furthermore, given the higher rate of type 2 diabetes in the indigenous population, we also having many indigenous patients. I know it’s hard to provide the same real life example to every trainee, so perhaps having a workshop with real life examples that really highlight the learning points is a good way to reach all trainees.”*
Enabling factors to learning about providing culturally responsive care	*“Education and exposure to seeing the differences. Most residents have a certain level of privilege, and are not aware of our own biases or privileges. Perhaps a rural or remote rotation could help unmask our own biases and privileges, and motivate change and desire to understand and learn more.”* *“Having clinical experiences that demonstrate a need for or gaps in their cultural competency. Hearing stories of people whose experience has been impacted by culturally insensitive care but also being provided concrete ways to improve this.”* *“I think we all want to be good at our job and provide effective care. Learning about shortcomings in our current healthcare system or practice through patient experience is a great motivator.”* *“I think a perls session dedicated to this topic would emphasize the importance. I think just through experience you realize how important it is to provide this cultural responsive care because your patients are more likely to take their medications, follow instructions and understand the disease process if you develop a culturally sensitive rapport.”* *“In general, this is something we need to learn. We have all taken care of patients at risk of exclusion or suffering discrimination and seeing that motivates us to get better.”* *“Understanding from a patient’s perspective how providing culturally responsive care helps patients achieve better health outcomes.”* *“Staff dedication to providing culturally responsive care, and receiving positive feedback about providing culturally responsive care (rather than just positive feedback about good clinical work/knowledge).”*
Barriers to learning about providing culturally responsive care	*“Topics that are not considered “medicine” are often not taken seriously by residents - as seen by relatively lower attendance for the half day sessions that are “soft skills”.”* *“I think residents and fellows are often overwhelmed with a. Getting through a large amount of clinical tasks every day and b. Trying to understand physiology that things like this may take a back seat to the prior. I also think that the level of burn out and fatigue is so high amongst learners that any additional “learning” requirements can be seen onerous.”*
Medical school education on culturally responsive care	*“I was very happy with my education from UofT. University of Toronto had a longitudinal curriculum to address providing culturally responsive care - including lectures on providing care for LGBTQ+, Black, Indigenous, patients with disabilities, racialized populations, and economically disadvantaged populations. This varied from lectures, to CBLs, to small group workshops.”* *“We had several didactic lectures on providing care to various marginalized groups as well as patient panels and small group discussions. We also had optional opportunities to volunteer with marginalized groups.”* *“I attended voluntary workshops on various global health topics focus on indigenous history and took the BC based online indigenous culture module for HCPs. We had various lectures on responsive care (or similar topics with other terms used) occasionally.”* *“Most of the initiatives were medical student driven.”* *“Not much to be honest. A few sessions on LGBT but they were never that informative and maybe a session on Indigenous health but also not super helpful.”* *“Sadly, there was no formal education. Very little discussion happened at times around specific cases/families.”*

#### Hands-on Opportunities as effective teaching modalities

Hands-on opportunities, such as continuity clinics and immersive experiences focusing on caring for children from historically marginalized groups, were perceived as effective teaching modalities for their promotion of longitudinal learning and relationship building. Core residents and subspecialty residents/fellows appreciate continuity clinics as they provide direct clinical exposure that enables them to better understand the subtleties and nuances for effective ways of communicating. Longitudinal encounters also facilitate opportunities to follow up on advocacy efforts and build stronger relationships with patients of different backgrounds, which allows time for retrospection, reflection, and growth.

Furthermore, immersive experiences are valued, as they encourage relevant, in-the-moment, focused learning around care for a given population, alongside support from a supervising physician. These intimate encounters create opportunities to challenge personal biases and learn from both patients and care providers.

#### Enabling factors to learning about providing culturally responsive care

Factors that enable learning about culturally responsive care include hands-on working experience with diverse patient populations, patient anecdotes regarding challenges during previous healthcare encounters, and observed clinical interactions. Trainees value hearing directly from patients with lived experience, especially those who previously received inadequate care.

Within the clinical environment, trainees express interest in working with staff physicians dedicated to providing culturally responsive care and receiving specific feedback about providing culturally responsive care, rather than comments just focusing on clinical knowledge.

Additionally, trainees value focused teaching about local resources that support specific populations, such as Indigenous community centers in the neighborhood, to become informed on services to offer patients.

#### Barriers to learning about culturally responsive care

Time constraints within the busy clinical environment, burnout, and lack of exposure to diverse patient populations were identified barriers that prevented trainees from learning about providing culturally responsive care. Despite its perceived importance, pediatric trainees find it challenging to balance learning about providing culturally responsive care with managing a heavy workload and studying. Exhaustion and burnout from clinical responsibilities may prevent learners from seeking out additional learning opportunities.

Inadequate exposure to patients from historically marginalized populations, and the perception of EDI as a “soft skill,” may contribute to learners feeling less invested in participating in further EDI training.

#### Medical school education on culturally responsive care

Most trainees previously received educational sessions on culturally responsive care throughout their undergraduate medical degree; however, these were often in the form of online modules and didactic lessons, which were perceived as less effective teaching modalities. Further opportunities to gain more experience in providing culturally responsive care were often medical student driven and optional. For example, some individuals received additional EDI training through completing online courses or partaking in voluntary sexual health and global health initiatives.

## Discussion

In medicine, EDI encompasses both visible and invisible characteristics of the patient, such as gender, race, sexual orientation, disability, socioeconomic status, and religion across all healthcare settings. It is not an outcome, but a process that is influenced by social, political, economic, and geographical climate. The pursuit of equity and justice is cultivated through medical education and culturally responsive care to truly work alongside and optimize health outcomes for the patients that we serve [1, 7].

Our study was designed to assess the confidence and interest of pediatric trainees in providing culturally responsive care to children and youth from historically marginalized populations. While many pediatric trainees reported a lack of confidence in providing culturally responsive care, nearly all respondents were interested in learning these skills. Respondents identified direct clinical exposure through rotations, immersive experiences, and continuity clinics as effective teaching modalities for delivering EDI curricula. Enabling factors to learning about culturally responsive care included hands-on working experience, patient anecdotes, and observed clinical interactions, while identified barriers included time constraints, burnout, and lack of exposure to diverse patient populations. Providing culturally responsive care is especially important in pediatrics given physicians’ responsibility to establish early therapeutic relationships that build trust in the healthcare system and positively influence children’s entire life trajectories. More broadly, this study’s findings offer valuable insights for medical educators to utilize when creating and enhancing EDI curricula.

Our study was consistent with existing literature demonstrating that the majority of healthcare providers believed it was important to deliver culturally competent care [12, 19–21]. In contrast, our findings that participants lacked confidence in delivering culturally responsive care has not been clearly demonstrated in similar studies. For example, Shepherd et al.’s study, which explored the perspectives of 56 American healthcare professionals, revealed that many healthcare workers felt confident engaging in cross-cultural medical interactions. However, there was minimal acknowledgement of systemic approaches to ensuring culturally responsive care, including recognition of racism, power imbalances, and awareness of individuals’ personal biases. Notably, the healthcare professionals in Shepherd et al.’s study were no longer trainees, with 40/56 (71.4%) respondents reporting at least 5 years of healthcare experience [12]. Our study did not specifically explore the underlying reasons for participants’ lack of confidence in delivering culturally responsive care, which may be reflective of lack of EDI training and/or acknowledgement of the complexity of delivering quality patient care. This offers an opportunity for further investigation.

Participants perceived hands-on training opportunities, such as continuity clinics, as effective teaching modalities. Continuity of care has recognized benefits on pediatric patient health outcomes, including decreased Emergency Department visits and hospital admissions, management of chronic illnesses, as well as parental reports of improved care quality and coordination [22–26]. Furthermore, respondents reported time constraints in the clinical environment and burnout as barriers preventing participation in EDI curricula. This is consistent with previous research describing barriers to providing culturally responsive care to pediatric patients. Suurmond et al.’s qualitative study of pediatric oncology care providers (12 pediatric oncologists and 13 nurses) similarly reported time constraints and busy workload as contributors to decreased quality of care for children from racialized backgrounds. However, in contrast to our findings, multiple respondents also reported language differences as a significant barrier to delivering culturally responsive care [27].

Findings from this study will be used to inform the iterative development and implementation of a more robust EDI curricula for UofT pediatric residents and fellows, and guide the development of culturally responsive learning outcomes to be embedded into the curriculum. These learning outcomes may include embedding the principles of EDI in clinical placements, applying the cultural knowledge of diverse students and communities to guide curricular development, and developing relationships with peers and community partners [28]. Similar efforts to improve EDI curricula have been made at other pediatric residency programs, such as the University of California San Francisco and University of Washington School of Medicine [29–31]. In 2022, Mullett et al. conducted a needs assessment on 125 pediatric residents at the University of Washington, revealing minimal prior instruction around providing culturally responsive care. In response, the team created a novel 25-hour EDI curriculum consisting of monthly didactic sessions addressing EDI definitions, history, and microaggressions. Our study builds on Mullett et al.’s work by emphasizing participants’ interest in learning more about how to deliver culturally responsive care, and demonstrates trainees’ understanding of their own knowledge gaps. In line with our study’s findings on effective teaching modalities, Mullett et al. noted that residents suggested ​​more case-based learning, skills practice, and simulation, rather than didactic teaching sessions [29, 30]. Together, these findings emphasize the importance of hands-on and interactive teaching modalities in the delivery of EDI curricula.

There were several limitations to this study. First, this single-center study only surveyed pediatric trainees at UofT, and may not be fully generalizable to other institutions. As well, analyses may be limited by the relatively low survey response rate, which may have contributed to non-response bias. Given this lower response rate, open-ended responses in survey format, specifically, may not meet standards for robust qualitative analyses [32]. However, it is important to note that this is the largest pediatric training program in Canada, which may in part mitigate the low response rate. Additionally, the survey sent out was not a validated measure, and was not piloted to ensure content validity. In terms of content, the term “culturally responsive care” was not explicitly defined, although the pediatric residents and fellows at UofT undergo mandatory EDI and Allyship training. Though the survey was anonymous, there may have been an influence of social desirability in questions pertaining to interest and importance of culturally responsive care. The study included a disclaimer that children and youth can belong to multiple marginalized groups; however, the closed-ended survey format did not allow respondents to express their confidence in caring for patients who affiliate with multiple historically marginalized identities (e.g. a child who identifies as both Indigenous and disabled) or identities not specifically included. Future studies are needed to integrate questions on patients with intersectional identities. Lastly, while the respondent pool was consistent with recent pediatric residency program gender compositions (64.2% female among UofT residents and 72.4% female across American residency programs), [33, 34] most trainees in our study identified as White and heterosexual. This may reflect a broader lack of representation of individuals from equity-seeking groups within Canadian postgraduate training programs. A 2020 survey determined that, compared to the Canadian Census population, medical students were less likely to identify as Black (1.7% vs. 6.4%; p < 0.001) or Indigenous (3.5% vs. 7.4%; p < 0.001) [34]. Future research directions may include exploring undergraduate medical students’ attitudes towards providing culturally responsive care and perceptions of how best to teach EDI curricula. Given physicians’ responsibility to treat diverse and intersectional groups of patients, ongoing research is required to ensure postgraduate trainees receive training early in their career to provide culturally responsive care, an important step in ‘health equity for all.’

## Conclusion

This study emphasized that most pediatric trainees did not feel confident providing culturally responsive care to children and youth from historically marginalized communities; however, the majority of postgraduate trainees were interested in engaging in further EDI education. Opportunities for enhancing educational experiences were suggested by respondents, with a particular focus on protected time to engage in hands-on training experiences. These study findings will be utilized to further develop enhanced EDI training for UofT’s pediatric trainees beginning in the 2022–2023 academic year. Furthermore, these results will inform both undergraduate and postgraduate medical educators about learner attitudes around effective modalities and identified barriers in delivering EDI curriculum, ultimately enhancing existing EDI curricula within other medical institutions.

## Data Availability

The datasets used and/or analyzed during the current study are available from the corresponding author on reasonable request.

## Abbreviations

EDI: Equity, diversity, and inclusion
UofT: University of Toronto
PGY: Postgraduate Year
